# Draft genome sequences of 25 candidate biocontrol bacteria against *Phytophthora cactorum*

**DOI:** 10.1128/mra.00502-25

**Published:** 2025-08-11

**Authors:** Juliane Ferreira, Hans Rediers

**Affiliations:** 1 Laboratory for Process Microbial Ecology and Bioinspirational Management, Centre of Microbial and Plant Genetics, Leuven, Belgium; 2 Leuven Plant Institute, KU Leuven 26657, Leuven, Belgium; The University of Arizona, Tucson, Arizona, USA

**Keywords:** biocontrol, *Phytophthora cactorum*, Illumina sequencing, whole genome sequencing

## Abstract

To provide a biocontrol solution for managing the phytopathogen *Phytophthora cactorum*, bacteria were tested for antagonistic activity *in vitro* and *in planta*. This paper presents the draft genomes of 25 candidate biocontrol organisms, providing a solid foundation to decipher the underlying mechanisms of their antagonistic activity.

## ANNOUNCEMENT

*Phytophthora cactorum* is one of the most economically important phytopathogens in the world, causing significant annual yield losses across a wide range of crops (1). Chemical control is often inefficient, increases fungicide resistance, and is being phased out for its detrimental side effects (2–4). To support a more sustainable agriculture, the focus is set on biological control. In a recent study, 38 phylogenetically diverse bacteria were tested for their biocontrol potential against *P. cactorum* (J. Ferreira, B. Lievens, and H. Rediers, unpublished data). Among these, 25 bacterial strains were not fully sequenced yet.

Here, we report the draft genome sequences of 25 candidate biocontrol strains, consisting of 11 *Bacillus* spp., 3 *Pantoea* spp., 2 *Peribacillus* spp., 2 *Priesta* spp., and 7 *Pseudomonas* spp. These strains belong to the PME&BIM −80°C glycerol culture collection and were isolated in-house from various niches, including banana rhizosphere, sludge, whiskey barrel, nectar, and insects (Table 1).

**TABLE 1:** Summary of the assembly parameters of the 25 candidate biocontrol bacteria^*a*^

Strain	Species ID^*c*^	ANI%^*d*^	Origin	Isolation source	GenBank accession no.	SRA accession no.	No. of reads^*e*^	Avg coverage	No. of ctg	Largest ctg (bp)^*f*^	Genome assembly size (bp)	Ctg N50 (kbp)^*f*^	GC%	No. of CDS^*g*^	No. of tRNA	No. of rRNA	Contamination %^*h*^	Completeness %^*h*^
BW7P1	*Pseudomonas bananamidigenes*	97.07	Sri Lanka	Banana rhizosphere	JBNMSE000000000	SRX28306108	4,938,501	117	72	1,129,168	6,077,771	365.4	60.5	5,421	59	5	0.15	100
ST01.11_032	*Priestia megaterium*	96.97	Belgium	Sludge	JBNMSD000000000	SRX28306109	959,661	25	111	824,800	5,572,100	370.3	37.5	5,699	60	13	0.8	100
ST01.11_034	*Bacillus altitudinis*	98.45	Belgium	Sludge	JBNMSC000000000	SRX28306121	3,204,954	126	33	908,681	3,708,887	614.2	41.0	3,776	62	7	0.25	100
ST01.11_050	*Bacillus pumilus*	95.45	Belgium	Sludge	JBNMSB000000000	SRX28306126	2,241,284	88	50	862,170	3,682,460	616.5	41.5	3,719	63	9	0	100
ST01.11_055	*Bacillus toyonensis*	98.34	Belgium	Sludge	JBNMSA000000000	SRX28306127	7,097,656	121	141	635,469	5,900,098	318.2	35.0	5,981	70	6	0.21	100
ST01.11_056	*Bacillus subtilis*	98.55	Belgium	Sludge	JBNMRZ000000000	SRX28306128	4,095,536	172	84	1,084,249	4,095,536	1,044.8	44.0	4,216	67	7	0.08	100
ST01.11_057	*Peribacillus simplex*	91.07	Belgium	Sludge	JBNMRY000000000	SRX28306129	8,493,073	228	811	414,279	5,416,895	85.2	39.5	5,710	70	9	0.95	100
ST01.11_082	*Bacillus pumilus*	95.49	Belgium	Sludge	JBNMRX000000000	SRX28306130	7,886,892	308	54	956,109	3,722,690	643.7	41.5	3,771	64	8	0	100
ST01.11_096	*Priestia megaterium*	97.06	Belgium	Sludge	JBNMRW000000000	SRX28306131	6,532,029	168	209	1,224,908	5,669,09	708.6	37.5	5,916	77	15	1.13	100
ST01.11_112	*Peribacillus simplex*	94.52	Belgium	Sludge	JBNMRV000000000	SRX28306132	6,486,018	179	381	279,955	5,293,975	99.3	40.0	5,282	66	11	0.3	100
ST12.11_029	*Bacillus wiedmannii*	96.28	Belgium	Whiskey barrel	JBNMRU000000000	SRX28306110	7,538,873	189	221	470,079	5,898,176	81.3	35.0	5,999	66	6	0.2	100
ST12.14_237^*b*^	*Pseudomonas zeae*	97.68	Belgium	Cornflower nectar	JBNMRT000000000	SRX28306111	6,193,593	136	119	732,130	6,585,441	331.0	59.0	5,926	60	4	0.37	100
ST12.14_379^*b*^	*Pseudomonas zeae*	97.67	Belgium	Borage nectar	JBNMRS000000000	SRX28306112	11,227,064	247	63	611,397	6,566,488	331.0	59.0	5,876	61	4	0.37	100
ST12.14_392^*b*^	*Pseudomonas zeae*	97.66	Belgium	Borage nectar	JBNMRR000000000	SRX28306113	5,316,136	116	100	732,577	6,581,027	379.7	59.0	5,912	61	4	0.37	100
ST12.14_393	*Pantoea agglomerans*	98.44	Belgium	Borage nectar	JBNMRQ000000000	SRX28306114	8,127,762	244	200	602,208	4,828,456	470.9	55.0	4,567	68	8	0.08	100
ST12.14_520^*b*^	*Pseudomonas kitaguniensis*	96.54	Belgium	Helleborine nectar	JBNMRP000000000	SRX28306115	2,427,057	55	266	257,359	6,145,138	131.2	59.5	5,704	59	3	0.87	100
ST12.15_006	*Bacillus velezensis*	99.99	Belgium	*Aphidus ervi*	JBNMRO000000000	SRX28306116	5,316,153	197	218	766,952	3,912,410	422.4	46.5	3,836	71	10	0.57	100
ST12.15_014	*Pseudomonas shirazensis*	99.17	Belgium	*Aphidus ervi*	JBNMRN000000000	SRX28306117	9,986,911	274	73	450,008	5,247,258	171.5	61.5	4,807	66	4	0.05	100
ST12.15_023	*Pseudomonas fragariae*	94.96	Belgium	*Psylla pyri*	JBNMRM000000000	SRX28306118	4,498,446	103	365	510,199	6,296,113	190.2	59.0	5,678	56	5	0.22	100
ST14.12_094	*Pantoea agglomerans*	98.52	Belgium	Helleborine nectar	JBNMRL000000000	SRX28306120	7,745,336	228	183	530,366	4,889,562	225.1	55.0	4,576	62	8	0.02	100
ST14.12_205	*Pantoea agglomerans*	98.33	Belgium	Helleborine nectar	JBNMRK000000000.2	SRX28306119	8,879,989	278	298	246,398	4,791,170	66.1	55.13	4,557	52	3	0.55	99.99
ST14.14_008	*Bacillus altitudinis*	97.91	Belgium	Whiskey barrel	JBNMRJ000000000	SRX28306122	7,561,929	285	57	818,994	3,845,368	298.5	41.0	3,914	60	7	0.01	100
ST14.14_009	*Bacillus subtilis*	99.96	Belgium	Whiskey barrel	JBNMRI000000000	SRX28306123	4,819,543	171	45	1,057,479	4,083,235	1,043.3	43.5	4,199	70	13	0.01	100
ST14.14_013	*Bacillus wiedmannii*	96.25	Belgium	Whiskey barrel	JBNMRH000000000	SRX28306124	6,741,869	166	242	941,155	5,890,985	415.8	35.0	5,926	72	8	0.17	100
ST14.14_029	*Bacillus altitudinis*	98.00	Belgium	Whiskey barrel	JBNMRG000000000	SRX28306125	7,900,903	292	227	489,933	3,828,296	243.2	41.5	4,003	62	11	0.62	100

^*a*^ All the parameters described are for assemblies with contigs above 200 bp.

^*b*^ Reads decontaminated with the following taxids: 135623, 91347, 135614, 28211, and 2759 using Kraken2.

^*c*^ Species-level identification was done using multi-locus sequence typing (MLST) using the PubMLST interface.

^*d*^ The average nucleotide identity based on BLAST analysis (ANIb) was done against the reference strain of the species.

^*e*^ The number of raw reads.

^*f*^ ctg, contig.

^*g*^ CDS, coding sequence.

^*h*^ Completeness and contamination percentages were determined with checkm2 using the neural network model.

Genomic DNA was obtained from overnight bacterial pure cultures inoculated from a single colony and grown on Tryptone Soy Broth (Oxoid). The DNA extraction was achieved using the DNeasy UltraClean Microbial Kit (Qiagen) following the manufacturer’s instructions. The 151 bp paired-end read libraries were prepared for Illumina sequencing on the NovaSeq platform with Novogenes (UK) or Genomics Core (Belgium), roughly as described previously (5). All downstream sequence analyses were performed on the Galaxy platform (6), with default parameters. After the quality check of the raw reads with FastQC (v0.12.1, 7), they were filtered and trimmed with Trimmomatic (v0.36, 8). Contaminant reads originating from carry-over during sequencing were discarded based on their taxonomic identification with Kraken2 (v2.1.3, 9). Paired reads with a Phred score above 30 were assembled *de novo* using SPAdes (v3.15.4, 10). The completeness and quality of the assembly were assessed with CheckM2 (v1.0.2, 11) and QUAST (v5.3.0, 12), respectively. Strain ST14.12/205 exhibited a high contamination rate and was therefore decontaminated by binning the assembly using MaxBin2 (v2.2.7, 13). Reads were then mapped to the target bin with Bowtie2 (v2.5.3, 14), and the mapped reads were reassembled following the described pipeline. Gene prediction and functional annotation were achieved with PGAP (v6.10, 15). Species identification was achieved using the PubMLST interface (accessed in February 2025, 16) and confirmed by assessing the average nucleotide identity (ANI) with the reference genome of the closest identified species using FastANI (v1.3, 17).

Overall, the draft genomes' average coverage was 180× (25×–308×), with an average of 226 contigs over 200 bp, and N50 of 66.1 kb to 1,044 kbp. Genome sizes and GC% varied for the species of *Bacillus* (3.7–5.9 Mbp; 35–47%), *Pantoea* (4.8 Mbp; 55%), *Peribacillus* (5.3–5.4 Mbp; 40%), *Piestia* (5.6–5.7 Mbp; 38%), and *Pseudomonas* (5.3–6.6 Mbp; 59–62%).

The availability of draft genomes of the candidate biocontrol strains will contribute to future studies to decipher the molecular mechanisms underlying their antagonistic activity toward *P. cactorum* and other relevant plant pathogens. In addition, it allows studying niche-specific genetic differences in strains belonging to the same species but isolated from various sources.

## Data Availability

The draft genome assemblies have been deposited in GenBank, and the raw reads before decontamination have been deposited in SRA. The corresponding accession numbers are listed and linked in Table 1. All the data produced are part of the BioProject PRJNA1216553, hosted by the NCBI organization.
