# Correction for Ferreira and Rediers, “Draft genome sequences of 25 candidate biocontrol bacteria against *Phytophthora cactorum*”

**DOI:** 10.1128/mra.01248-25

**Published:** 2026-03-23

**Authors:** Juliane Ferreira, Hans Rediers

## AUTHOR CORRECTION

Volume 14, no. 9, e00502, 2025, https://doi.org/10.1128/mra.00502-25. Paragraph 2: “These strains belong to the PME&BIM −80°C glycerol culture collection and were isolated in-house from various niches, including banana rhizosphere, sludge, whiskey barrel, nectar, and insects…” should read “These strains belong to the PME&BIM and CMPG −80°C glycerol culture collection and were isolated from various niches, including banana rhizosphere, sludge, whiskey barrel, nectar, and insects…”

The strain *Pseudomonas bananamidigenes* BW7P1 originated from the Center of Microbial and Plant Genetics (CMPG) and was isolated in Sri Lanka in 1992 (K. Vlassak, I. Van Holm, L. Duchateau, J. Vanderleyden, et al., Plant Soil 145:51–63, 1992, https://doi.org/10.1007/BF00009541). The genome sequence was previously released as “*Pseudomonas* sp. BW7P1” in 2022 under accession GCF_024981055.1.

